# Canine obesity in Portugal: perceptions on occurrence and treatment determinants

**DOI:** 10.1186/1751-0147-57-S1-P8

**Published:** 2015-09-25

**Authors:** Rita Payan-Carreira, Teresa Sargo, Maria Manuel Nascimento

**Affiliations:** 1 School of Agrarian and Veterinary Sciences, Universidade de Trás-os-Montes e Alto Douro, Vila Real, Portugal; 2 Animal and Veterinary Research Center, CECAV, Universidade de Trás-os-Montes e Alto Douro, Vila Real, Portugal; 3 Didactics and Technology in Teacher Education, University of Aveiro, Aveiro, Portugal

## Introduction

Pet obesity is a leading health threat worldwide; its real numbers remain unclear in Portugal; still, the perception exits that obesity is common in routine practice, often ignored by dog owners.

## Objectives

We performed a survey for practising veterinarians in Portugal mainland to establish some dog obesity-related parameters.

## Methods

The “on-line” simplified questionnaire focused on the obesity prevalence and characterization of obese dogs, owner recognition and compliance to weight-loss plans, and contributing factors to the success of overweight control. Twenty-seven complete surveys were considered, covering veterinary hospitals (9) and practices (18), representing ≈32.500 dogs/year, in a male/female proportion close to 1.

## Results

Obesity prevalence was 40% (5-80%). For obese dogs, owners seldom requested counselling (13%) or weight control measures (12%). When a diet plan was proposed (74%) only 12% of the dogs were engaged. The 6 most frequently referred breeds were Labrador, Mongrels, Poodle, English Cocker, Golden Retriever and French Bulldog. Obesity was more frequent in females than males, and in spayed dogs. Severe obesity was less frequent than overweight or moderate obesity. Owners of severely obese dogs were often elderly, but middle-aged in moderately obese dogs. Owner gender was unimportant for the obesity degree and the compliance with treatment, though motivation to a weight-loss plan was higher in younger ones. The study showed that weight-loss programs had low success in moderate (46%) and severe (36%) obesity, which might resulted from economic and social/cultural issues or the owner lifestyle.

## Conclusion

This survey gave important cues for factors affecting the success of obesity treatments in Portuguese dogs.

